# Retention in care, resource utilization, and costs for adults receiving antiretroviral therapy in Zambia: a retrospective cohort study

**DOI:** 10.1186/1471-2458-14-296

**Published:** 2014-03-31

**Authors:** Callie A Scott, Hari S Iyer, Kelly McCoy, Crispin Moyo, Lawrence Long, Bruce A Larson, Sydney Rosen

**Affiliations:** 1 Center for Global Health and Development, Boston University, Crosstown Center, 3rd Floor, 801 Massachusetts Avenue, Boston, MA 02118, USA; 2 Zambia Center for Applied Health Research and Development, Plot 4649 Beit Road, Rhodes Park, P.O. Box 30910, Lusaka 10101, Zambia; 3 Zambian Ministry of Health, Ndeke House, P.O. Box 30205, Lusaka 10101, Zambia; 4 Vanderbilt Institute of Global Health, Vanderbilt University, 2525 West End Avenue, Suite 750, Nashville, TN 37203, USA; 5 Health Economics and Epidemiology Research Office, Wits Health Consortium, Department of Clinical Medicine, School of Internal Medicine, Faculty of Health Sciences, University of the Witwatersrand, Helen Joseph Hospital, Perth Road, Westdene, Johannesburg 2092, South Africa; 6 Department of International Health, School of Public Health, Boston University, Crosstown Center, 3rd Floor, 801 Massachusetts Avenue, Boston, MA 02118, USA

**Keywords:** HIV/AIDS, Adult, Antiretroviral therapy, Resource-limited setting, Costs, Outcomes, Zambia

## Abstract

**Background:**

Of the estimated 800,000 adults living with HIV in Zambia in 2011, roughly half were receiving antiretroviral therapy (ART). As treatment scale up continues, information on the care provided to patients after initiating ART can help guide decision-making. We estimated retention in care, the quantity of resources utilized, and costs for a retrospective cohort of adults initiating ART under routine clinical conditions in Zambia.

**Methods:**

Data on resource utilization (antiretroviral [ARV] and non-ARV drugs, laboratory tests, outpatient clinic visits, and fixed resources) and retention in care were extracted from medical records for 846 patients who initiated ART at ≥15 years of age at six treatment sites between July 2007 and October 2008. Unit costs were estimated from the provider’s perspective using site- and country-level data and are reported in 2011 USD.

**Results:**

Patients initiated ART at a median CD4 cell count of 145 cells/μL. Fifty-nine percent of patients initiated on a tenofovir-containing regimen, ranging from 15% to 86% depending on site. One year after ART initiation, 75% of patients were retained in care. The average cost per patient retained in care one year after ART initiation was $243 (95% CI, $194-$293), ranging from $184 (95% CI, $172-$195) to $304 (95% CI, $290-$319) depending on site. Patients retained in care one year after ART initiation received, on average, 11.4 months’ worth of ARV drugs, 1.5 CD4 tests, 1.3 blood chemistry tests, 1.4 full blood count tests, and 6.5 clinic visits with a doctor or clinical officer. At all sites, ARV drugs were the largest cost component, ranging from 38% to 84% of total costs, depending on site.

**Conclusions:**

Patients initiate ART late in the course of disease progression and a large proportion drop out of care after initiation. The quantity of resources utilized and costs vary widely by site, and patients utilize a different mix of resources under routine clinical conditions than if they were receiving fully guideline-concordant care. Improving retention in care and guideline concordance, including increasing the use of tenofovir in first-line ART regimens, may lead to increases in overall treatment costs.

## Background

Of the estimated 800,000 adults living with HIV in Zambia in 2011, roughly half were receiving antiretroviral therapy (ART) [1,2]. The Government of Zambia revised national HIV treatment guidelines for adults in 2007 to recommend the use of tenofovir as a standard component of first-line ART [3]. Guidelines were revised again in 2010 to raise the CD4 cell count threshold for ART eligibility from 200 cells/μL to 350 cells/μL and again in 2013 to remove any CD4 cell count threshold for ART eligibility for pregnant women [4,5]. Each of these changes substantially increased either the number of patients eligible for treatment or the cost of the drugs that comprise the treatment.

As treatment scale up continues, and as the Government of Zambia considers further guidelines changes that could expand ART eligibility even more or affect the quality of care for patients already on ART, information about the actual care provided to patients after initiating ART in Zambia can help guide decision making. Published papers have reported on the positive clinical and programmatic outcomes for patients initiating ART in Zambia [6,7] and on the costs of providing ART in Zambia [8,9], but no published papers have described both the actual care provided by the public sector clinics and hospitals that serve the vast majority of patients and the associated costs at the patient level. Our objective was to estimate retention in care, the quantity of resources utilized, and costs for adults initiating treatment under routine clinical conditions in Zambia.

## Methods

### Analytic overview

We enrolled a retrospective cohort of HIV-infected adults who initiated ART at six treatment sites in Zambia between July 2007 and October 2008, after tenofovir replaced stavudine in national guidelines as a standard component of first-line antiretroviral therapy. We collected patient-level data on retention in care and resource utilization from outpatient medical records. We estimated site- and country-level data on unit costs from financial reports, procurement records, and other sources. We estimated the proportion of patients retained in care, the average quantity of resources utilized per patient and per patient retained in care, and the average cost per patient and per patient retained in care through one year after ART initiation at all six sites and through two and three years after ART initiation at two of the six sites (where additional data were available at the time of data collection). We included resources utilized and costs incurred at the treatment site only; off-site resource utilization and costs were excluded. Costs were calculated from the provider’s perspective in 2011 US dollars.

### Study sites

Large scale, public sector provision of ART in Zambia began in Lusaka in 2004 and rapidly expanded. At the time of this study, clinics and hospitals across the country were providing ART, laboratory tests, and medications for opportunistic infections to patients free of charge. We purposively selected six of these sites to illustrate different models or settings for adult ART delivery in Zambia (Table 1). Sites included two primary health clinics in Lusaka Province (sites 1 and 2), a primary health clinic in Copperbelt Province (site 3), a second-level general hospital in Western Province (site 4), a first-level district hospital in Southern Province (site 5), and a second-level mission hospital in Southern Province (site 6). The number of active patients enrolled in the ART program at each site in 2008 ranged from 524 at site 5 to 5,748 at site 4.

**Table 1:** Study site characteristics

**Characteristic**	**Site 1**	**Site 2**	**Site 3**	**Site 4**	**Site 5**	**Site 6**
Province	Lusaka	Lusaka	Copperbelt	Western	Southern	Southern
Facility type	Primary health clinic	Primary health clinic	Primary health clinic	Second-level general hospital^a^	First-level district hospital^a^	Second-level mission hospital^a^
Setting	Urban	Urban	Urban	Urban	Urban	Rural
Number of active patients enrolled in ART program, 2008	5,488	5,102	1,167	5,748	524	2,981

ART: antiretroviral therapy.

^a^First-level hospitals are district-level hospitals. Second-level hospitals are larger, provincial-level or general hospitals.

At the time of this study, ART eligibility, recommended ART regimens, and schedules for laboratory and clinical monitoring were in accordance with the 2007 national HIV treatment guidelines [3]. When CD4 testing was available, patients were eligible to initiate ART if they had (1) a CD4 < 200 cells/μL, (2) a CD4 < 350 cells/μL with a WHO clinical stage 3 disease, or (3) a WHO clinical stage 4 disease. When CD4 testing was not available, patients were eligible to initiate ART if they had (1) a WHO clinical stage 3 or 4 disease or (2) a WHO clinical stage 2 disease and a total lymphocyte count <1200 cells/μL. Guidelines recommended a first-line ART regimen containing tenofovir, emtricitabine, and either efavirenz or nevirapine, with abacavir and lamivudine substituted for tenofovir and emtricitabine for patients with low creatinine clearance. Guidelines also recommended at least two CD4, hemoglobin, white blood cell count, and blood chemistry tests and at least five clinic visits during the first year on ART.

### Sample enrollment

Adults who initiated ART at least 12 months prior to data collection at sites 1, 3, 4, and 5, and at least 36 months prior to data collection at sites 2 and 6, were selected consecutively from clinic registers and enrolled in the study. Data collection at sites 2 and 6 occurred after data collection at the other sites. Patients at sites 2 and 6 therefore had a longer follow-up period than patients at the other sites because additional data were available at the time of data collection.

Patients were eligible for enrollment if they were at least 15 years of age at the time of ART initiation and were not known to have transferred to another clinic during the study follow up period. The target enrollment at each site was 150 patients. Fewer patients were enrolled at site 5 due to low patient volume. Seven patients, one each at sites 1, 2, 4, and 5, and three at site 3, were excluded after enrollment because they were found not to meet all study inclusion criteria. Total enrollment was therefore 846 patients.

### Ethics statement

The Boston University Medical Center Institutional Review Board and the University of Zambia Biomedical Research Ethics Committee provided ethical approval of the study (protocol numbers H-28282 and 003-06-07). A waiver of informed consent was granted by both committees because the study was a retrospective review of routinely collected information from patient medical records.

### Data collection

Data on patient outcomes and resources utilized at the treatment site during the first 12 or 36 months following ART initiation were obtained from each study patient’s medical record. All resources used by the provider to deliver outpatient care to study patients were included, even if the resource cost was borne by another entity. Data on the costs of resources utilized were collected from site-level financial records, interviews with site managers, and country-level drug price lists and procurement records.

### Classification of patient outcomes

Study patients at all sites were assigned outcomes at 12 months after initiating ART. Patients at sites 2 and 6 were also assigned outcomes at 24 and 36 months after initiating ART. Patients were classified as *known to have died* if a confirmation of death was noted in their medical record before the 12-, 24-, or 36-month endpoint. Patients were classified as *lost to follow up* if they were ≥3 months late for their last scheduled consultation or medication pickup before the endpoint but had no confirmation of death in their medical record. Patients not classified as *known to have died* or *lost to follow up* were classified as *retained in care*.

### Estimation of unit costs

We used previously published methods to estimate unit costs for fixed and variable resources utilized at the treatment sites by study patients, as outlined in Table 2[10-13]. Fixed resources included buildings, equipment, and support staff employed in the ART clinic who do not see patients. Variable resources included ARV drugs, non-ARV drugs, laboratory tests, and provider time for clinic visits.

**Table 2:** Methods for estimating unit costs

**Resource**	**Method**
***Fixed resources***	
Buildings and equipment	Estimated upfront investment costs using a replacement cost approach, then calculated annualized costs using a 3% annual discount rate and an estimated working life of 5 years for equipment and 50 years for buildings [11]. Annual building and equipment costs for the ART clinic at each site were divided by the total number of active HIV patients at the site per year to estimate a cost per patient-year in care. The total number of active HIV patients at the site was calculated by summing the number of ART patients at the site with the number of non-ART patient-equivalents weighted based on a ratio of the average number of patient visits to the site per year for non-ART versus ART patients.
Support staff	Estimated annual cost of support staff employed in the ART clinic at the study sites during the study period based on 2011 salaries and allowances. The proportion of staff time allocated to ART versus non-ART activities was based on staff estimates. Annual support staff costs were divided by the total number of active HIV patients at the site per year to estimate a cost per patient-year in care. The total number of active HIV patients at the site was calculated by summing the number of ART patients at the site with the number of non-ART patient-equivalents weighted based on a ratio of the average number of patient visits to the site per year for non-ART versus ART patients. Costs for higher-level administrative support staff, including staff based in the health facility in which the ART clinic was located, were excluded from the analysis.
***Variable resources***	
ARV drugs	Estimated as the average per unit cost for all units of a particular drug purchased for the Zambia national HIV program in 2011, or during the most recent year available if no units of a particular drug were purchased in 2011, as recorded in the Global Price Reporting Mechanism [12]. Data on ART regimen dispensed were used to determine the appropriate drug formulation (fixed dose tablet, single dose tablet) for each ARV drug dispensed [3].
Non-ARV drugs	Estimated from standard Zambian Ministry of Health per package costs [13].
Laboratory tests	For sites 3, 4, 5, and 6, where laboratory tests are run onsite, costs were estimated as the sum of unit costs for reagents, consumables, equipment, labor, and space. Costs of reagents and consumables were estimated from standard Zambian Ministry of Health per package costs [13]. Annual laboratory equipment, labor, and space costs were divided by the total number of laboratory tests performed per year to estimate a per test cost at each site. For sites 1 and 2, where laboratory tests are run at a centralized laboratory, costs were provided by the Centre for Infectious Disease Research in Zambia Central Laboratory in Lusaka and included the cost of reagents, consumables, equipment, and labor used for each test (Henry Latner, personal communication, April 29, 2011).
Provider time for clinic visits	Estimated the total annual cost of staff time for each type of provider conducting patient consultations. The proportion of staff time allocated to ART versus non-ART activities was based on staff estimates. Cost per visit was calculated by dividing the total cost of staff time for each type of provider, valued at 2011 salaries and allowances, by the total number of patient consultations with each provider type per calendar year.

Costs estimated in Zambian Kwacha (ZMK) were first adjusted to 2011 levels, if necessary, using the consumer price index and then converted to US dollars at a rate of 4,861 ZMK/$, the average exchange rate for 2011 [14,15]. We excluded costs of resources used for inpatient care and resources used for outpatient care above the level of the treatment site, such as government or NGO costs of oversight or training. We also excluded costs of resources procured by individual patients, such as transport to the clinic, and costs of resources used prior to ART initiation.

### Average resource utilization and costs

We calculated the total quantity of each resource utilized by each patient and then calculated total costs for each patient by multiplying the unit cost for each resource by the total quantity utilized. We calculated average resource utilization and costs by dividing total resource utilization and costs for all patients by the total number of patients in the cohort. We repeated the same calculations for the subset of patients retained in care 12 months after initiating ART. At sites 2 and 6, where we had 36 months of follow up for each patient, we also calculated the average annual cost for the subset of patients retained in care 24 and 36 months after initiating ART by estimating the average cost for these patients over the first two or three years on ART and then dividing by two or three years.

We calculated average resource utilization and costs for each of sites 1 through 6 alone, as well as for sites 1 through 6 combined. The confidence intervals for the means for the six sites combined were adjusted for clustering at the site level (using the cluster option with the regression command in Stata).

## Results

### Cohort characteristics

Patients initiated ART at a median age of 35 years and a median CD4 cell count of 145 cells/μL; 60% of patients were female (Table 3). The majority of patients (59%) initiated on regimens containing tenofovir while the remainder initiated on zidovudine (14%), abacavir (14%), and stavudine (13%). ART regimen at initiation varied widely by site.

**Table 3:** Cohort characteristics

**Characteristic**	**Site 1**	**Site 2**	**Site 3**	**Site 4**	**Site 5**	**Site 6**	**All sites**
Total sample size	149	149	147	149	102	150	846
Follow up time, months	12	36	12	12	12	36	---
Median age at ART initiation, years [IQR]	35 [30–40]	35 [30–43]	34 [29–40]	33 [29–42]	35 [31–41]	37 [31–46]	35 [30–42]
Sex, % female	63	52	68	66	45	58	60
Median CD4 at ART initiation, cells/μL [IQR]^a^	149 [97–211]	127 [67–206]	136 [84–199]	150 [77–237]	140 [74–273]	160 [106–209]	145 [86–212]
Regimen at ART initiation, % of patients							
TDF-containing regimen	36	86	80	15	54	80	59
AZT-containing regimen	30	5	13	4	30	7	14
ABC-containing regimen	0	5	0	76	0	0	14
d4T-containing regimen	34	5	7	5	16	13	13

ART: antiretroviral therapy; ABC: abacavir; AZT: zidovudine; d4T: stavudine; IQR: interquartile range; TDF: tenofovir.

^a^793 of 846 patients in the sample had a CD4 cell count at initiation.

### Patient outcomes

One year after ART initiation, 75% of patients were retained in care, 11% were known to have died, and 15% were lost to follow up (Table 4). The proportion of patients retained in care ranged from 69% to 78% depending on site.

**Table 4:** Retention in care one year after initiating antiretroviral therapy

**Patient outcome, n (%)**	**Site 1**	**Site 2**	**Site 3**	**Site 4**	**Site 5**	**Site 6**	**All sites**
Retained in care	116 (78)	103 (69)	109 (74)	116 (78)	76 (75)	113 (75)	633 (75)
Known to have died	13 (9)	23 (15)	14 (10)	7 (5)	6 (6)	26 (17)	89 (11)
Lost to follow up	20 (13)	23 (15)	24 (16)	26 (17)	20 (20)	11 (7)	124 (15)

At sites 2 and 6, where patients were followed for three years after ART initiation, retention in care decreased from 69% and 75% one year after initiating ART to 64% and 72% two years after initiating ART and 61% and 64% three years after initiating ART. For patients retained in care one year after initiating ART at sites 2 and 6, the probability of being retained in care two years after initiating ART was 93%. For patients retained in care two years after initiating ART, the probability of being retained in care three years after initiating ART was 92%. The proportion of patients known to have died was 18% two and three years after initiating ART.

### Resource utilization

During the first year after ART initiation, patients utilized, on average, 9.1 months’ worth of NRTI drugs and 9.0 months’ worth of NNRTI drugs or protease inhibitor (PI) drugs (Table 5). Patients also utilized, on average, 5.2 months’ worth of co-trimoxazole, 1.2 CD4 tests, 1.1 blood chemistry tests, 1.2 full blood count tests, 5.4 clinic visits with a doctor or clinical officer, and 7.9 visits with a pharmacist.

**Table 5:** Average quantity of resources utilized per patient during the first year on antiretroviral therapy

**Resource**	**Site 1**	**Site 2**	**Site 3**	**Site 4**	**Site 5**	**Site 6**	**All sites**
***Quantity of resources utilized per patient for total sample***							
ARV drugs, number of patient-months							
NRTI combinations							
TDF 300 mg + FTC 200 mg	3.7	7.6	7.3	1.3	4.1	8.8	5.6
AZT 300 mg + 3TC 150 mg	2.6	0.0	0.8	0.5	2.2	1.0	1.1
ABC 300 mg + 3TC 150 mg	0.1	0.9	0.0	6.4	0.0	0.1	1.3
d4T 30 mg + 3TC 150 mg	2.5	0.6	0.6	0.5	1.4	1.1	1.1
Any NRTI combination	8.9	9.1	8.8	8.7	7.8	11.1	9.1
NNRTI or PI							
NVP 200 mg	6.4	7.7	4.0	2.5	3.7	4.0	4.8
EFV 600 mg	2.2	1.5	4.5	6.2	4.0	6.8	4.2
LPV/r 200/50 mg	0.1	0.0	0.0	0.0	0.0	0.0	0.0
Any NNRTI or PI	8.8	9.2	8.5	8.6	7.7	10.8	9.0
Non-ARV drugs, number of patient-months^a^							
Co-trimoxazole 400/80 mg	6.4	8.8	1.3	3.2	0.2	9.8	5.2
Laboratory tests, number of tests^b^							
CD4 test	1.5	1.3	1.4	0.5	1.5	1.2	1.2
Blood chemistry test^c^	0.2	1.9	1.0	0.3	1.0	2.2	1.1
Full blood count^d^	1.3	1.2	1.0	0.4	2.0	1.3	1.2
Clinic visits with each type of provider, number of visits^e^							
Doctor or clinical officer	5.0	5.3	5.3	3.4	6.6	7.4	5.4
Nurse	7.4	6.8	5.4	4.2	6.6	7.4	6.3
Counselor	9.2	9.2	6.1	7.6	6.6	6.6	7.6
Pharmacist	9.5	10.0	6.1	7.2	6.6	7.3	7.9
Fixed resources, number of patient-months^f^	9.9	9.3	9.6	10.1	9.6	9.8	9.7
***Quantity of resources utilized per patient for subset of sample retained in care one year after initiating ART***							
ARV drugs, number of patient-months							
NRTI combinations							
TDF 300 mg + FTC 200 mg	4.3	10.2	9.3	1.6	5.1	10.8	6.9
AZT 300 mg + 3TC 150 mg	3.3	0.0	1.0	0.6	2.9	1.3	1.5
ABC 300 mg + 3TC 150 mg	0.1	1.2	0.0	7.6	0.0	0.1	1.6
d4T 30 mg + 3TC 150 mg	3.1	0.9	0.8	0.5	1.7	1.4	1.4
Any NRTI combination	10.9	12.2	11.1	10.4	9.6	13.6	11.4
NNRTI or PI							
NVP 200 mg	7.9	10.3	5.1	2.9	4.6	5.1	6.0
EFV 600 mg	2.6	2.0	5.6	7.4	5.0	8.1	5.2
LPV/r 200/50 mg	0.2	0.0	0.0	0.0	0.0	0.0	0.0
Any NNRTI or PI	10.7	12.3	10.7	10.2	9.6	13.2	11.2
Non-ARV drugs, number of patient-months^a^							
Co-trimoxazole	7.9	11.8	1.6	3.8	0.3	12.1	6.5
Laboratory tests, number of tests^b^							
CD4 test	1.8	1.9	1.6	0.6	1.7	1.5	1.5
Blood chemistry test^c^	0.1	2.5	1.0	0.3	1.0	2.6	1.3
Full blood count^d^	1.6	1.6	1.0	0.5	2.2	1.6	1.4
Clinic visits with each type of provider, number of visits^e^							
Doctor or clinical officer	5.9	6.6	6.4	3.8	8.0	8.7	6.5
Nurse	8.8	8.6	6.5	4.8	8.0	8.7	7.5
Counselor	11.1	11.9	7.4	8.9	8.0	7.8	9.2
Pharmacist	11.4	13.0	7.4	8.5	8.0	8.6	9.5
Fixed resources, number of patient-months^f^	12.0	12.0	12.0	12.0	12.0	12.0	12.0

3TC: lamivudine; ABC: abacavir; ART: antiretroviral therapy; ARV: antiretroviral; AZT: zidovudine; d4T: stavudine; EFV: efavirenz; FTC: emtricitabine; LPV/r: ritonavir-boosted lopinavir; NNRTI: non-nucleoside reverse transcriptase inhibitor; NRTI: nucleoside reverse transcriptase inhibitor; NVP: nevirapine; PI: protease inhibitor; TDF: tenofovir.

^a^Patients in our sample received other non-ARV drugs in addition to co-trimoxazole. These included, but were not limited to: multivitamins, folic acid supplements, ferrous sulfate supplements, paracetamol, fluconazole, ibuprofen, and amoxicillin.

^b^In addition to CD4 tests, blood chemistry tests, and full blood counts, patients in our sample also received <0.1 on average of each of the following lab tests during the first year on ART: acid-fast bacillus, hepatitis B test, malaria test, pregnancy test, rapid plasma reagin test, and viral load test.

^c^A blood chemistry test could include any combination of the following tests: creatinine, aspartate aminotransferase, alanine aminotransferase, total bilirubin, direct bilirubin, and urea.

^d^A full blood count includes hemoglobin and a white blood count, among other counts.

^e^A single clinic visit for patients in our sample could include a consultation with more than one type of provider (doctor or clinical officer, nurse, counselor, or pharmacist).

^f^Fixed resources included buildings and infrastructure, equipment, supplies, vehicles, and staff time for staff employed in the ART clinic who do not see patients.

As expected, patients retained in care one year after initiating ART utilized substantially more resources than those known to have died or lost to follow up, including 11.4 months’ worth of NRTIs and 11.2 months’ worth of NNRTIs or PIs, 6.5 months’ worth of co-trimoxazole, 1.5 CD4 tests, 1.3 blood chemistry tests, 1.4 full blood count tests, 6.5 clinic visits with a doctor or clinical officer, and 9.5 visits with a pharmacist. The quantity of each resource utilized varied widely by site.

### Unit costs

ARV drug costs, which were constant across sites, varied by drug regimen (Table 6). Abacavir and tenofovir were more expensive than zidovudine and stavudine, and efavirenz was more expensive than nevirapine. The cost of co-trimoxazole was modest compared to the cost of ARV drugs.

**Table 6:** Unit costs (in 2011 USD) for resources utilized by adults receiving antiretroviral therapy

**Resource**	**Site 1**	**Site 2**	**Site 3**	**Site 4**	**Site 5**	**Site 6**
ARV drugs, cost per patient-month dispensed						
NRTI combinations						
TDF 300 mg + FTC 200 mg	10.54	10.54	10.54	10.54	10.54	10.54
AZT 300 mg + 3TC 150 mg	8.41	8.41	8.41	8.41	8.41	8.41
ABC 300 mg + 3TC 150 mg	18.64	18.64	18.64	18.64	18.64	18.64
d4T 30 mg + 3TC 150 mg	3.01	3.01	3.01	3.01	3.01	3.01
NNRTI or PI						
NVP 200 mg	2.34	2.34	2.34	2.34	2.34	2.34
EFV 600 mg	4.26	4.26	4.26	4.26	4.26	4.26
LPV/r 200/50 mg	36.70	36.70	36.70	36.70	36.70	36.70
Non-ARV drugs, cost per patient-month dispensed						
Co-trimoxazole 400/80 mg	0.93	0.93	0.93	0.93	0.93	0.93
Laboratory tests, cost per test^a^						
CD4 test	2.87	2.87	11.90	10.56	18.16	10.06
Blood chemistry test	2.93	2.93	5.01	7.23	5.11	2.27
Full blood count	2.80	2.80	2.14	5.24	5.60	1.11
Clinic visits with each type of provider, cost per visit^b^						
Doctor or clinical officer visit	1.71	1.82	0.57	0.37	2.86	1.01
Nurse visit	0.52	1.01	0.48	0.40	1.91	0.59
Counselor visit	0.56	0.15	0.31	1.00	2.11	2.21
Pharmacist visit	0.42	0.32	0.29	0.45	1.50	0.28
Fixed resources, cost per month in care^c^	1.03	1.17	1.21	0.60	6.04	3.31

3TC: lamivudine; ABC: abacavir; ARV: antiretroviral; AZT: zidovudine; d4T: stavudine; EFV: efavirenz; FTC: emtricitabine; LPV/r: ritonavir-boosted lopinavir; NNRTI: non-nucleoside reverse transcriptase inhibitor; NRTI: nucleoside reverse transcriptase inhibitor; NVP: nevirapine; PI: protease inhibitor; TDF: tenofovir; USD: United States Dollar.

^a^Laboratory tests for sites 1 and 2 were done at a centralized, high volume laboratory in Lusaka. Laboratory tests for sites 3, 4, 5, and 6 were done onsite.

^b^Clinic visit costs include the cost of staff time for staff who see patients.

^c^Fixed costs include the cost of buildings and infrastructure, equipment, supplies, vehicles, and staff time for staff employed in the ART clinic who do not see patients.

Laboratory test costs varied widely between sites. The cost per CD4 test, for example, ranged from $2.87 at sites 1 and 2, where tests were run at a centralized, high-volume laboratory in Lusaka, to $18.16 at site 5 where tests were run onsite at a relatively low-volume laboratory. The cost per clinic visit with each type of provider and the cost of fixed resources per month in care also varied widely between sites.

### Average cost per patient

During the first year after ART initiation, the average cost per patient for the total sample was $198 (95% CI, $157-$239), ranging from $151 (95% CI, $137-$165) at site 1 to $251 (95% CI, $229-$273) at site 5 (Table 7). The average cost per patient for the subset of the sample remaining in care one year after initiating ART was $243 (95% CI, $194-$293), ranging from $184 (95% CI, $172-$195) at site 1 to $304 (95% CI, $290-$319) at site 5.

**Table 7:** Average costs and cost breakdown for the first year on antiretroviral therapy

	**Site 1**	**Site 2**	**Site 3**	**Site 4**	**Site 5**	**Site 6**	**All patients at all sites**^**d**^
***Average cost, 2011 USD (95% CI)***							
Average cost per patient for total sample	151 (137–165)	185 (168–202)	166 (154–179)	209 (192–225)	251 (229–273)	242 (224–260)	198 (157–239)
Average cost per patient for subset of sample retained in care one year after initiating ART	184 (172–195)	245 (235–254)	205 (198–212)	247 (233–261)	304 (290–319)	296 (285–307)	243 (194–293)
***Breakdown of average cost per patient for subset of sample remaining in care one year after initiating ART, 2011 USD (% of total)***							
ARV drugs^a^	123 (67)	166 (68)	148 (72)	207 (84)	116 (38)	178 (60)	159 (66)
Non-ARV drugs^a^	12 (6)	20 (8)	4 (2)	5 (2)	0 (0)	22 (7)	11 (5)
Laboratory tests	11 (6)	18 (7)	27 (13)	11 (5)	49 (16)	23 (8)	22 (9)
Clinic visits^b^	26 (14)	27 (11)	11 (5)	17 (7)	67 (22)	33 (11)	28 (11)
Fixed resources^c^	12 (6)	14 (6)	15 (7)	7 (3)	72 (24)	39 (13)	24 (9)
Total	184 (100)	245 (100)	205 (100)	247 (100)	304 (100)	296 (100)	243 (100)

ART: antiretroviral therapy; ARV: antiretroviral; CI: confidence interval; USD: United States Dollar.

^a^Unit costs for ARV and non-ARV drugs were standardized across all sites so variation in total ARV and non-ARV drug costs between sites are due to differences in utilization.

^b^Clinic visits include the cost of staff time for staff who see patients.

^c^Fixed costs include the cost of buildings and infrastructure, equipment, supplies, vehicles, and staff time for staff employed in the ART clinic who do not see patients.

^d^Because the sample size varied by site, the average cost for all patients at all sites is slightly different than the average cost for all sites.

At sites 2 and 6, where patients were followed for three years after ART initiation, the average annual cost per patient remaining in care decreased from $245 (95% CI, $235-$254) and $296 (95% CI, $285-$307) one year after initiating ART to $233 (95% CI, $221-$245) and $272 (95% CI, $262-$282) two years after initiating ART and $221 (95% CI, $208-$234) and $270 (95% CI, $260-$279) three years after initiating ART. Decreases in the cost per patient remaining in care over time are due primarily to decreases in the quantity of resources utilized over time.

Antiretroviral drugs were the largest cost component at all six sites, comprising between 38% (site 5) and 84% (site 4) of the average cost per patient for the subset of the sample remaining in care one year after initiating ART.

## Discussion

The Government of Zambia revised national HIV treatment guidelines in 2007 to recommend the use of tenofovir as a standard component of first-line ART and in 2010 and 2013 to expand eligibility criteria for patients not yet on ART [3-5]. As the Government of Zambia considers new guidelines changes, with potentially large implications for program planning and budgets, information on the actual care provided under previous guidelines can help guide decision making. We used primary, patient-level data to estimate retention in care, the quantity of resources utilized, and the costs of providing ART to adults in routine clinical practice in Zambia after adoption of the 2007 national guidelines.

Patients initiated ART at a median CD4 cell count of 145 cells/μL; 59% initiated on a tenofovir-containing regimen. One year after ART initiation, 75% of patients were retained in care. Patients retained in care received, on average, 11.4 months’ worth of ARV drugs, 1.5 CD4 tests, 1.3 blood chemistry tests, 1.4 full blood count tests, and 6.5 clinic visits with a doctor or clinical officer. The average cost for all patients over the first year after ART initiation was $198 (95% CI, $157-$239) while the average cost per patient retained in care one year after ART initiation was $243 (95% CI, $194-$293). Both the average quantity of resources utilized and unit costs varied widely by site, resulting in average costs per patient retained in care ranging from $184 (95% CI, $172-$195) at site 1 to $304 (95% CI, $290-$319) at site 5.

Our findings have several implications for program planning. First, as has been widely reported throughout Africa, Zambian patients are initiating ART late in the course of disease progression [16]. The median CD4 cell count at initiation for patients in our study, 145 cells/μL, was considerably lower than the 200 cells/μL threshold for ART eligibility in place at the time when these patients initiated ART [3]. This suggests that many patients may have reported to care long after meeting the eligibility criteria for ART initiation. Future guidelines changes that increase the CD4 threshold for ART eligibility, and expand eligibility to a larger proportion of the HIV-infected population, may have a smaller impact than anticipated, both in terms of increased health benefits and increased program costs, if patients are not effectively identified and linked to care soon after meeting eligibility criteria.

Second, a large proportion of patients (25%) are no longer in care within one year of initiating ART and attrition continues, at a slower rate, through three years after initiating ART. The average cost per patient in our sample for the first year on ART was $198. If we were to estimate the average cost to produce a patient retained in care one year after initiating ART, calculated as the sum of all costs for all patients divided by just the number of patients remaining in care, it would be $265. If all patients remained in care, we would expect an average cost per patient of $243, greater than the average cost per patient in the study of $198, but less than the cost to produce a patient remaining in care of $265.

Third, actual resource utilization varied considerably from what would be expected if patients were receiving fully guideline-concordant care. At the time of this study, guidelines recommended initiation on a regimen containing tenofovir, emtricitabine, and either efavirenz or nevirapine. Only 59% of patients in our sample initiated ART on a tenofovir-containing regimen while the remaining 41% of patients initiated ART on regimens containing zidovudine, abacavir, or stavudine. Patients in our sample initiated ART between July 2007 and October 2008, soon after tenofovir became the recommended first-line ART regimen in Zambia. It is not known whether study sites were still in the process of adopting tenofovir at the time of this study or if a similar mix of regimens would be seen among patients initiating ART at the same sites today. Nevertheless, these deviations from expected, guideline-concordant utilization months after guidelines were changed are notable.

Regardless of ART regimen at initiation, patients retained in care one year after initiating ART received, on average, fewer than the 12 months’ worth of ARV drugs required to ensure uninterrupted treatment. These patients also received only 1.5 CD4 tests, 1.3 blood chemistry tests, and 1.4 full blood count tests during their first year on ART. Guidelines recommend two CD4 tests, at least two blood chemistry tests, and at least two hemoglobin and white blood cell count tests, components of a full blood count test, during the first year after ART initiation [3]. While patients utilized fewer drugs and laboratory tests than they would have under fully guideline-concordant care, they had more frequent clinical consultations than recommended by guidelines. Patients retained in care had an average of 6.5 visits with a doctor or clinical officer and 9.5 visits with a pharmacist during their first year on ART, compared to the five visits recommended in the guidelines [3]. All of these examples suggest that it may not be appropriate to assume that guidelines and practice will be identical for the purposes of program planning and budgeting.

The proportion of patients retained in care one year after ART initiation in our study, 75%, is comparable to other published estimates of retention in care during the first year on ART [16]. Our estimated cost of $243 per patient retained in care one year after ART initiation is lower than, but still comparable to, previously published estimates of the cost per patient-year of ART in Zambia. Tagar et al. estimated an average cost of $278 per patient-year of ART (in 2010–2011) for a 30-site sample by estimating total facility-level costs at each site during the study period and dividing by an estimated number of patient-years in care at each site during the same period [17]. Bratt et al. estimated a cost per patient-year of ART ranging from $278 to $523 (in 2008 US dollars) by estimating unit costs from a 12-site sample and applying the unit costs to a set of resources that patients would be expected to utilize during their first year on ART under idealized clinical care conditions [9]. Marseille et al. estimated a cost of $428 per patient-year of ART in on-site costs (in 2010 US dollars) for the average facility in their 45-site sample by applying unit costs to a mix of site- and patient-level data on resource utilization [8]. The cost per patient-year of ART was $638 when off-site costs were included. Our study builds on these previous cost estimates by: (1) providing insight into the actual quantity of resources utilized and services received per patient at the level of the treatment site, and (2) differentiating between patients retained in care and those not retained.

Our study has several limitations. First, results are from six sites so we cannot attribute variation in costs of care between sites to specific site-level characteristics. These sites were purposively selected to capture variation in location and size. While results from these sites provide insight into standard types of treatment sites in Zambia, they are not necessarily representative of the ART treatment program in Zambia as a whole. Second, because patients were only classified as *lost to follow up* if they were ≥3 months late for their last scheduled consultation or medication pickup at the study endpoint, we likely underestimate true attrition during the study follow up period. Third, patient outcomes in this analysis are limited to what could be ascertained from a retrospective review of medical records. For patients no longer attending the study clinic, we could not always distinguish between those who had transferred to another clinic, died, or been lost to follow up due to incomplete records. Third, we excluded resource utilization and costs associated with inpatient care and outpatient care received prior to ART initiation. We also excluded costs incurred by the patient and costs for program management above the facility level. Cost estimates from Marseille et al., who found that only $428 of the $638 in total costs per patient-year of treatment were incurred on site, suggest that this exclusion of costs above the facility level may lead to a significant underestimate of the total cost of providing ART at the treatment sites in our study sample. Finally, results reflect retention in care, resource utilization, and costs for patients who initiated ART in 2007 and 2008, after the adoption of tenofovir as a standard component of first-line ART in 2007 but prior to the adoption of new HIV treatment guidelines in 2010 that increased the CD4 threshold for ART eligibility from 200 cells/μL to 350 cells/μL and the announcement in 2013 that Zambia would provide life-long ART to all pregnant women regardless of CD4 cell count [4,5]. Both changes may lead to a higher median CD4 cell count at initiation which could, in turn, result in changes in retention in care, resource utilization, and costs.

## Conclusions

In summary, adult patients in Zambia initiate ART late in the course of disease progression and a large proportion drop out of care within one year. Unit costs for each resource and the average quantity of resources utilized vary widely by treatment site, as do the resulting average costs of care, suggesting opportunities for efficiency gains. Overall, patients utilize a different mix of resources under routine clinical conditions than if they were receiving fully guideline-concordant care. The differences between guidelines and practice highlight the importance of looking at what is actually happening, and not just what is expected to happen, to ensure effective program planning and accurate budgeting. Improving retention in care and guideline concordance, including increasing the use of tenofovir in first-line ART regimens, may lead to increases in overall treatment costs.

## Competing interests

The authors declare that they have no competing interests.

## Authors’ contributions

LL and SR designed the study. CAS and KM oversaw data collection. CAS, HSI, and KM analyzed the data. CAS, HSI, KM, CM, LL, BAL, and SR assisted with interpretation of results. CAS wrote the first draft of the manuscript. CAS, HSI, KM, CM, LL, BAL, and SR reviewed and edited the manuscript. All authors read and approved the final manuscript.

## Pre-publication history

The pre-publication history for this paper can be accessed here:

http://www.biomedcentral.com/1471-2458/14/296/prepub
